# A Sensor Array for the Measurement of Relative Motion in Lower Limb Prosthetic Sockets

**DOI:** 10.3390/s19122658

**Published:** 2019-06-12

**Authors:** Veronika Noll, Sigrid Whitmore, Philipp Beckerle, Stephan Rinderknecht

**Affiliations:** 1 Institute for Mechatronic Systems in Mechanical Engineering, Technische Universität Darmstadt, Otto-Berndt-Str. 2, 64287 Darmstadt, Germany; beckerle@ims.tu-darmstadt.de (P.B.); rinderknecht@ims.tu-darmstadt.de (S.R.); 2 Virginia Polytechnic Institute and State University, Blacksburg, VA 24061, USA; 3 Elastic Lightweight Robotics Group, Robotics Research Institute, TU Dortmund University, 44227 Dortmund, Germany

**Keywords:** relative movement, lower limb prosthetics, biomechanic measurement tasks, quantifying socket fit, gait analysis

## Abstract

The relative motion between residual limb and prosthetic socket could be a relevant factor in quantifying socket fit. The measurement of these movements, particularly in dynamic gait situations, poses a challenging task. This paper presents the realization of a measurement concept based on multiple optical 2D-motion sensors. The performance of the system was evaluated on a test rig considering accuracy and precision as well as accomplished measurement frequency and reliability of the system. Additionally, results of a pilot study measuring the relative motion between residual limb and prosthetic socket at seven specific locations of one individual with transtibial amputation during straight level walking are presented. The sensor functionality of the array was confirmed and the test rig experiments were comparable to the previously tested functional model ($err_{\mathrm{rel}}=0.52\pm 1.87$%). With a sampling frequency of 1.3 kHz to be distributed among the number of sensor units, the developed system is suitable for investigating the relative movement between residual limb and prosthetic socket in dynamic gait situations. Results of the pilot study show the majority of relative motion occurring during the second half of the gait cycle. The measured relative motions show the residual limb sinking deeper into the socket, extending in the Sagittal plane and rotating internally in the Transverse plane during stance phase. Data captured during swing phase indicate a lower limb extension in the Sagittal plane as well as an external rotation in the Transverse plane.

## 1. Introduction

The socket is the mechanical interface between prosthesis and residual limb. It accounts for stability, ensures control over the prosthetic device, and determines its level of comfort. Thus, stump–socket interaction strongly influences the well-being and mobility of amputees [1]. To ensure the quality of socket fit, quantitative measures are advantageous. Pistoning or relative movement between residual lower limb and prosthesis is considered to be one indicative parameter of socket fit quality [2].

The most common techniques that are used to acquire data on the relative movement between residual limb surface and prosthetic socket are: motion capture [3,4,5,6], other optical means [7,8], inductive sensors [9,10,11,12], and vacuum pressure fluctuations of elevated vacuum suspension systems [11,13]. These approaches are subject to different drawbacks. For instance, vacuum pressure fluctuations cannot be used to evaluate the relative motion at specific or problematic locations within the interface. Additionally, despite the number of different measurement approaches, studies presenting gait cycle dependent data of relative motion during ambulation are scarce [3,4,9,14].

A concept for measuring the relative motion between residual limb surface and prosthetic socket at specific locations in dynamic gait situations is presented in [15]. The proposed concept is based on optical 2D-motion sensor units, whose applicability has been tested experimentally using a functional model consisting of one sensor unit on a test rig as well as in biomechanical substitute studies [16].

This paper presents the subsequently realized low-cost measurement system capable of interfacing with an array of sensors. The implementation of the optimized measurement chain is presented in Section 2. In Section 3, the measurement system is evaluated experimentally on a test rig regarding accuracy, precision, achievable sampling frequency, and overall reliability. A pilot study using seven sensors to measure the relative motion between residual limb and prosthetic socket is reported in Section 4. Based on a discussion of the results, the paper is concluded in Section 5 where an outlook to future work is also given.

## 2. Measurement System

**Figure 1:**
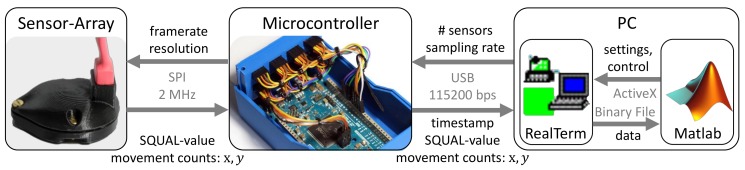
Realized measurement chain with indication of communication between system components: sensor array (**left**); microcontroller (**center**); and software running on PC (**right**).

A schematic of the system components and measurement chain is shown in Figure 1. The system consists of up to eight sensor units and an electronics box containing a microcontroller connected to a PC via USB.

A breakout version (https://www.tindie.com/products/jkicklighter/adns-9800-laser-motion-sensor/) of the ADNS-9800 optical sensor and accompanying ADNS-6190 lens (Avago Technologies, Broadcom Ltd., San Jose, CA, USA) was chosen due to its high-end specifications (30 g, 3.8 m/s, programmable maximum 12,000 fps and 8200 cpi). The sensor compares sequentially acquired images to mathematically derive resolution-dependent movement counts for the relative displacement along its two main axes *x* and *y*. Each sensor unit is protected by a case, which also serves as a mounting base for attachment to a prepared location on a prosthetic socket or to a testing bench. Both the protective case and mounting base are fabricated in polylactide (PLA) with a Fused-Deposition-Modeling 3D-printer. The sensors interface with the microcontroller over a Serial-Peripheral-Interface (SPI) connection.

The Arduino Due (Arduino AG, Ivrea, Italy) was chosen as a replacement for the Arduino Uno (Arduino AG, Ivrea, Italy) used in the functional model [16] due to its higher processor and SPI frequencies (64 MHz and up to 42 MHz) as well as compatibility with the existing firmware. It utilizes the sensor’s burst mode register reading functionality to continuously acquire movement counts in the sensor’s main axes *x* and *y* along with the surface quality (SQUAL-) value from the sensor. The SQUAL-value is a dimensionless value equal to 1/4 of the features observed by the sensor and is an indicator of surface texture. The microcontroller transmits these quantities and a timestamp in μs to a PC via a USB connection at a specified sampling frequency.

Incoming data are received and saved to a binary file by the serial terminal program RealTerm (https://realterm.sourceforge.io/). The measurement process is controlled from a Matlab (MathWorks, Natick, MA, USA) GUI, from which the number of sensors, sampling frequency per sensor and calibration factor are set. The calibration factor is calculated from uncalibrated measurements over a known distance. After completion of a measurement, the GUI converts movement counts into displacement distance with Δd as the displacement in mm, $c_{x}$ the movement counts, and $k_{x}$ a dimensionless, surface-dependent calibration factor:

(1) $$\Delta d\;=\;k_{x}c_{x}\frac{25.4\;\mathrm{mm}/\mathrm{inch}}{8200\;\mathrm{cpi}}.$$

Optimization of the measurement chain with respect to sampling rate is achieved by minimizing register reading times, on-board processing, and the number of bytes transmitted to the PC. The sensor resolution and frame rate are set to their maximum values of 8200 cpi and 12,000 fps. The SPI frequency was set to 2 MHz and the baud rate to 115,200 bps. These modifications increase the system’s top frequency from about 62.5 Hz for one sensor unit [16] to approximately 1.3 kHz, to be distributed among all attached sensors.

## 3. Test Rig Evaluation

The following section presents the methodology and results of the test rig evaluation of an array consisting of four sensor units.

### 3.1. Methodology

**Figure 2:**
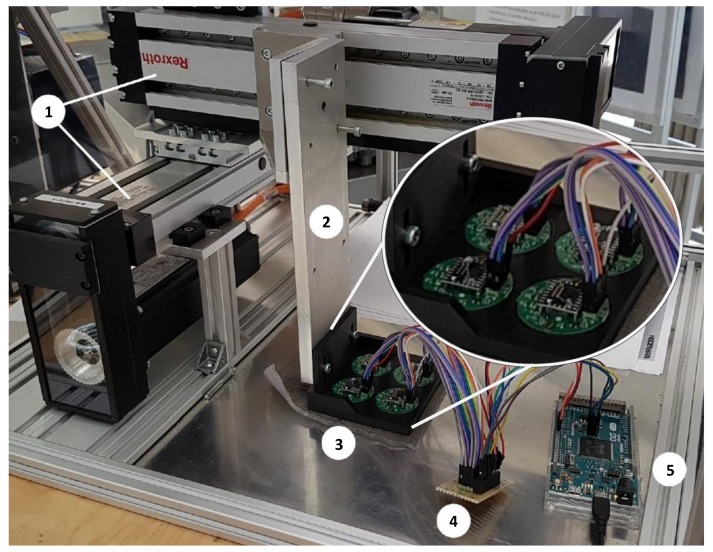
The test rig with linear drives (**1**); cantilever (**2**); test bracket, sensors and liner (**3**); wiring junction pcb (**4**); and Arduino Due (**5**) with USB cable leading to laptop; adapted from [17].

The system’s performance was evaluated on a bi-axial test rig. Two linear drives (Indradyn, Bosch Rexroth, Lohr am Main, Germany) were mounted perpendicular to one another. Motion was controlled by a Rexroth MTX 13V programmable open-loop control system. The rig moved a cantilever in the horizontal plane over a base plate. A bracket with four attached sensors was mounted to the cantilever and a patch of liner material placed beneath. In this testing phase, a wiring junction printed circuit board (pcb) was used to connect the sensor units to the Arduino. The described measurement setup is shown in Figure 2.

The sampling rate was set to 200 Hz per sensor, which meets the capabilities of commercially-available products for gait analysis (≥50 Hz). Measurements of uni-axial and diagonal motion, each consisting of fifteen motion steps, were conducted for each combination of distance (1, 5, 10, and 40 mm) and velocity (1, 10, and 100 mm/s). Complete measurement data (*x* and *y* movement counts, SQUAL-value, and timestamp) were continuously recorded. Movement counts were converted to displacements and scaled with the chosen calibration factor. The sections of each measurement containing motion were automatically extracted and the total displacement recorded for each step determined. The relative error with respect to the known displacement of the test rig Δx was determined with Δd, as determined in Equation (1):

(2) $$err_{\mathrm{rel}}\;=\;\frac{\Delta d\;-\;\Delta x}{\Delta x}.$$

### 3.2. Performance Results

**Figure 3:**
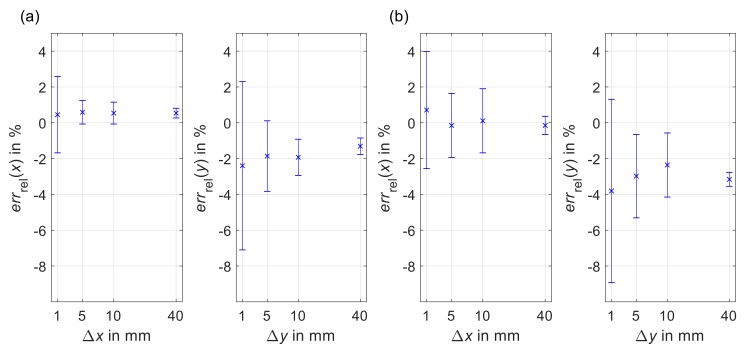
Mean (cross marker) and standard deviation (error bar) of the relative error for one sensor from (**a**) uni-axial and (**b**) diagonal movement. Velocities were combined at each distance. Calibration factors were $k_{x}$ = 2.018 and $k_{y}$ = 1.987.

Results are presented using data of a single sensor of the four-sensor array as a proxy for the behavior of the overall system. The calculated relative errors are shown in Figure 3. The bias of the mean (cross marker) indicates accuracy while the standard deviation (error bar) represent precision. Because of the limited influence of test rig velocity on sensor performance [16], velocities were combined for each distance. A bias of less than 0.9% was achieved for the *x*-direction at 40 mm. The *y*-direction achieved biases within 1.43% for uni-axial motion and 4.76% for diagonal motion at 40 mm. The standard deviation at 40 mm was less than 0.9% for uniaxial as well as diagonal movements.

The calculated relative errors demonstrate comparable results in both accuracy and precision to the functional model [16]. Although the behavior among sensors was comparable, differences were noted in the required calibration factors with values for the four sensors ranged between 1.976 and 2.018 for $k_{x}$ and between 1.915 and 1.987 for $k_{y}$. As the thickness of the liner patch varied by approximately 1 mm along the diagonal, these differences likely stemmed from offset variations between the individual sensors and the liner. The system’s effective resolution depends upon the necessary calibration factor and is in the low micrometer range (the chosen sensor resolution of 8200 cpi corresponds to 3.1 μm per count).

Sampling frequencies per sensor were confirmed for four sensors between 25 Hz and 275 Hz in 25 Hz increments. Across all frequencies, the difference between set sampling period and the average sampling period calculated from the recorded timestamps was 0.01 ± 0.15 μs.

Errors were found to occur only rarely. Out of 839 measurements, seven cases of transient behavior in both *x* and *y* displacements were recorded. Since this error affected only individual sensors and not all four, the source was unlikely to have been due to external factors, e.g., a bump to the test-rig. These errors were easily identifiable and localized, i.e., they did not affect subsequently recorded data. In cases where measurement duration approached or exceeded ten minutes, data recording at the PC-end was delayed, resulting in a loss of data if the measurement was ended too soon after the period of interest. Given that the duration of measurements is intended to be around two minutes for different dynamic walking tasks, this problem is unlikely to be relevant in practice.

### 3.3. Indication

The developed measurement system accommodates up to eight sensors and has a maximum single-sensor sampling frequency of 1299 Hz. Considering all four tested sensor units, experimental evaluations show the system’s functionality to be $err_{\mathrm{rel}}=-0.34\pm 1.28$% in uniaxial and $err_{\mathrm{rel}}=-0.90\pm 0.98$% in diagonal test-rig motion. Measurement errors were found to be uncommon, of known types, and not critical to the measurement.

## 4. Pilot Study

This section presents the methodology and results of the conducted pilot study.

### 4.1. Methodology

The pilot study was conducted with a single participant, an active male (K4, 69 years, 1.88 m, 90 kg) with transtibial amputation on the right side (stump length 11 cm, amputated for five years, Pro Flex XC foot (Össur hf, Reykjavik, Iceland)) who is wearing a custom-built measurement socket based on a hydrostatic plaster impression with pin lock mechanism and size 30 silicone Relax${}^{™}$ Locking liner (Össur hf, Reykjavik, Iceland). Prior to the experiment, the participant provided his written informed consent. The study was conducted with a positive vote by the ethics committee of the Technische Universität Darmstadt (reference number: EK40/2017) and is in accordance with the Declaration of Helsinki in its current version.

The developed measurement system was used to record the relative motion at seven specific socket locations. The sampling frequency was set to 100 Hz per sensor unit. Measurement sites were identified via semi-structured interviews with the participant and the prosthetist. Four of the chosen sites have previously been experienced as problematic and can mostly be assigned to anatomical landmarks (e.g., distal end of residual bones). In addition, three unproblematic socket locations were chosen for comparison. Table 1 summarizes the positions of the individual sensor units.

For a seamless sensor unit integration into the socket, the identified sites were already taken into account during the socket manufacturing process: The positive residual limb plaster model with marked locations was digitized with the 3D-scanner ATOS III (GOM GmbH, Braunschweig, Germany), individual sensor base plates corresponding to the plaster model surface at the measurement locations were designed, and 3D-printed in PLA using the Ultimaker 2+ (Ultimaker B.V., Geldermalsen, Netherlands). These sensor base plates were attached to the plaster model, on which the carbon socket was then fabricated.

**Figure 4:**
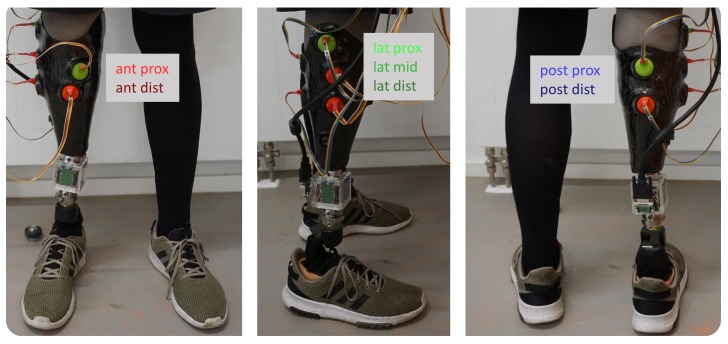
Participant equipped with custom-built measurement socket and load adapter.

In addition to the presented measurement system, a custom-built measurement adapter [18] integrated into the prosthetic structure was used to measure the loads at the distal end of the socket with a sampling frequency of 500 Hz. The two systems were synchronized by a triggering signal provided by the adapter. Figure 4 shows the participant wearing the custom-built measurement socket with integrated sensor array as well as the adapter for measuring the occurring loads.

For this pilot study, the participant completed a 5 m walkway of straight level walking 15 times. Data of the two measurement systems were interpolated to 200 Hz. The measured force in proximodistal (pd) direction was used to automatically identify and isolate completed gait cycles (GC). Of these gait cycles, the first and last steps of each measurement were excluded. In total, 54 gait cycles remained for data analysis.

### 4.2. Results and Discussion

**Figure 5:**
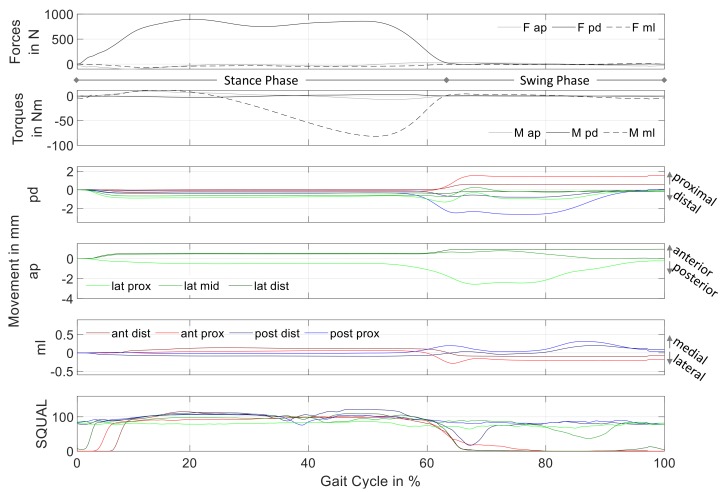
Mean of measurement data of 54 gait cycles; from top to bottom: forces, torques, movement in proximodistal, anteroposterior, mediolateral direction, and SQUAL-values sensor units. The colors used for the sensor units correspond to Figure 4.

For reasons of clarity, Figure 5 shows the mean of all 54 gait cycles of the measurement data. The first two subplots summarize the external loads measured by the adapter, while the other subplots show data captured by the sensor array. Due to the different measurement sites, all sensors captured the relative motion in pd-direction, while anteroposterior (ap) and mediolateral (ml) movement was only detected by sensor units affixed in the Sagittal and Coronal plane, respectively. The bottom subplot shows the SQUAL-values of each sensor unit.

The movement results show almost no motion during stance phase. This changes with the decrease of $F_{\mathrm{pd}}$ at around 55% of the gait cycle: With less weight bearing on the prosthesis, the relative motion between residual limb and prosthetic socket increased. Most of the relative motion was detected during swing phase. Movements in ml-direction were of smaller magnitude compared to motion in the Sagittal plane.

Except for three sensor units in the first 5–10% of the gait cycle, SQUAL-values were relatively constant during stance phase. More changes could be observed during swing phase: Three sensor units (lat dist, ant prox, and ant dist) showed a SQUAL-value of 0 for the majority of swing phase. The SQUAL-values of two sensor units (post dist and lat mid) showed a brief break-in during the first and second half of swing phase, respectively.

As stated in Section 2, the SQUAL-value is equal to 1/4 of the features the sensor uses to calculate its movement over the reference surface. Consequently, a SQUAL-value of zero means that the sensor cannot detect any features for movement calculation. This most likely stems from an out of bounds distance between sensor and reference surface (liner), which corresponds to movement of the residual limb normal to the sensor lens. During these time instants, the sensor does not continue to measure movement in its main axes. Thus, the shown movement data of the affected sensor units were underestimated for lower SQUAL-values (break-in during swing phase) and inconsequential at time instants with SQUAL-values of zero.

The small movement during stance phase might indicate the tight socket fit. With load bearing in the longitudinal direction, the residual limb is pressed radially against the socket wall. The resulting normal force between residual limb surface and socket wall leads to a high friction force, which prevents most of the relative movement during stance phase. This relatively constant position of the limb within the socket during stance phase has been observed previously [9]. Nevertheless, observed relative movements during stance phase show a uniform behavior in pd-direction while movement observations in ap- and ml-direction depend on the measuring site. Overall, the recorded movement of the residual limb inside the socket during stance phase might be described as sinking deeper into the socket, extending in the Sagittal plane and rotating internally in the Transverse plane.

The detected relative motion at the different socket locations during swing phase is mostly non-uniform: While negative motion in pd-direction is noted at measuring site post prox, the movement detected at ant prox is in the opposite direction. This might indicate a rotation of the socket relative to the residual limb in the Sagittal plane, which would correspond to the shank extending inside the socket. Compared to these two measuring sites, there is little movement detected in pd-direction at the other sites. In ap-direction, most relative motion is detected at lat prox in the first half of swing phase. Corresponding to the residual limb moving in posterior direction, this matches the shank extending inside the socket. The data are in accordance with [4], which reports residual limb extension inside the socket in the first half of swing phase. In addition to having a smaller magnitude compared to the other directions, the observed movement in ml-direction at the two posterior measurement sites is qualitatively the same, with a maximum at about 90% of the gait cycle. Jointly with the movement indication in the opposite direction at ant prox (60–70% of GC), this might correspond to the shank performing an external rotation in the Transverse plane within the socket during swing phase.

**Figure 6:**
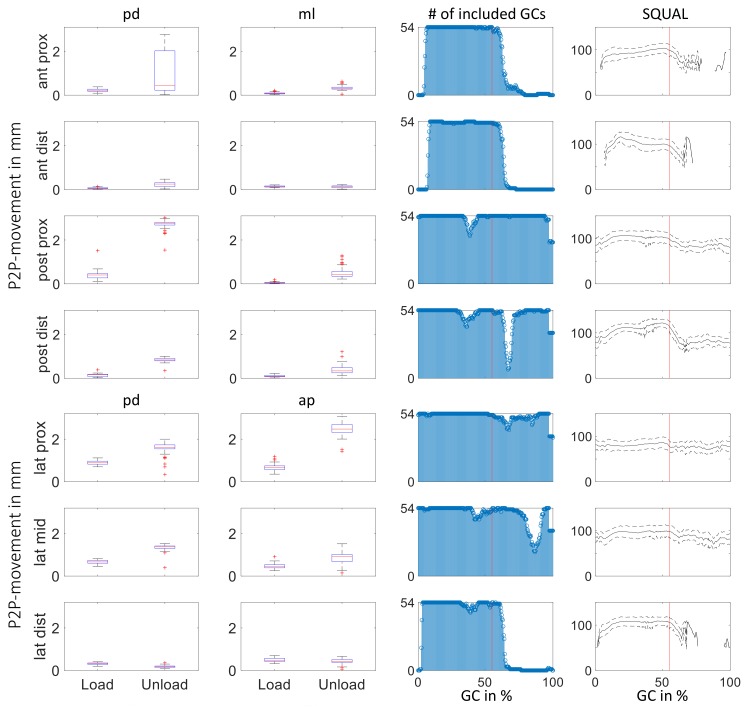
Analysis of peak-to-peak values at the different measuring sites. The data of sites are arranged according to location on anatomical planes: Coronal plane (first four rows) and Sagittal plane (following three rows). The first two columns summarize peak-to-peak values of relative motion in 54 gait cycles in the two anatomical directions corresponding sensor location (pd and ml for Coronal plane, and pd and ap for Sagittal plane). The two columns on the right indicate the data basis underlying peak-to-peak calculations.

Figure 6 summarizes peak-to-peak (P2P) values measured during the loading (0–55% GC) and unloading (55–100% GC) phase of each gait cycle at the different sites as box plots. The first four rows show the movement of the residual limb inside the socket in the Coronal plane, while the last three rows depict movement in the Sagittal plane. Contrary to data shown in Figure 5, only measurement data of each time instant captured at a corresponding SQUAL-value between 50 and 150 were included in the analysis. The third column shows the subsequent number of included gait cycles at each instant of time. Additionally, mean ± standard deviation of SQUAL-values of included data are shown in the rightmost column. Vertical lines indicate to which box plot (load vs. unload) the data belong.

The magnitude of relative motion between residual limb and prosthetic socket is quite small; no peak-to-peak values above 3.1 mm are recorded. Except for relative motion at lat dist, more relative motion is recorded during the unloading phase (last 45% of GC) compared to the loading phase (first 55% of GC). A tendency of peak-to-peak values being of greater magnitude during unloading phase compared to loading phase as well as for proximal measurement sites compared to those located more distally was observed.

Taking into account the distal fixation of the liner within the socket (pin lock system) and assuming a constant strain $\epsilon \;=\;\frac{\Delta l}{l_{0}}$ along the pd axis of the liner led to higher peak-to-peak relative motion at proximal compared to distal measuring sites ($l_{0,\mathrm{prox}}$ > $l_{0,\mathrm{dist}}$).

At the two anterior measurement sites as well as at lat dist, the collected movement data are of improvable quality during swing phase; the number of included gait cycles at the three sites was reduced from over 50 at 60% GC to less than 15 at 65% of GC. Time instants of the gait cycle without mean SQUAL-values indicate where the affected sensor units did not detect the necessary features for movement data calculation. At post dist and lat mid, a dip was observed in the number of included gait cycles at 65% and 95% of GC, respectively.

Peak-to-Peak movement data recorded during swing phase by the sensor units on the anterior side of the socket and at lat dist need to be interpreted with caution. The shown values only consider data of parts of the loading and unloading phase (compare SQUAL in rightmost column), which might not include maximal movements. Excluded data at 95% of GC would most likely not affect P2P calculation.

As stated previously, variations in SQUAL-value might stem from too much movement normal to the measurement plane of the corresponding sensor unit. Quantification of the dependency of SQUAL-values on measurement distance between sensor unit and reference surface as well as influence on displacement data might lead to the extension of usable sensor data information.

Compared to most studies quantifying residual limb socket peak-to-peak displacements of transtibial amputees using a socket with pin-lock suspension during walking [3,4,9,12], the detected movement within the pilot study was relatively small. A study investigating the variation of sock thickness on peak-to-peak relative motion shows a distinct decrease as sock thickness increased and vice versa [12]. These findings support the hypothesis of a tight socket fit being responsible for the small magnitudes of detected movement (particularly during stance). Another study presents P2P displacements normal to the socket wall ranging between 0.7 and 9.4 mm and varying for participants as well as measuring sites [10]. The data (see [10] ([Figure 7])) show a tendency of movements of greater magnitude at distal measuring sites compared to measuring sites placed most proximally. Additionally, a comparison of P2P displacements at the proximal measuring sites show consistently greater magnitudes on the anterior side. This supports the hypothesis of residual limb movement normal to the socket wall being responsible for SQUAL-values of 0 at the measuring sites ant prox and ant dist (as well as lat dist).

## 5. Conclusions

Overall, the presented data of the pilot study are biomechanically plausible and in accordance with prior findings of studies analyzing residual limb movement within the sockets of individual with transtibial amputation during gait [4,9,10]. Compared to other measuring approaches [4,11,13], the presented measuring system has an additional value as it enables the analysis of relative motion at a number of specific measuring sites, thus facilitating the detection of compliant soft tissue behavior.

Being tethered constrains the area of use of the system; there is potential for removing this impediment by switching to either wireless data transmission or on-board data storage. Nevertheless, even in its current state, the presented measuring system facilitates the analysis of relative movement between residual limb and prosthetic socket at specific measurement sites for different dynamic gait situations. Future studies will include the exploration and analysis of different gait situations, socket manipulations, as well as prosthetic components and their impact on relative motion. Additionally, the distribution of relative motion with respect to problematic and unproblematic areas will be investigated.

## Figures and Tables

**Table 1:** Identified measurement sites.

Location	Group	Description	Abbreviation
anterior proximal	comparison	medial tibial flair	ant prox
anterior distal	problem	distal end of tibia bone	ant dist
lateral proximal	comparison	lateral support	lat prox
lateral middle	problem	fibula	lat mid
lateral distal	problem	distal end of fibula bone	lat dist
posterior proximal	comparison	center of posterior compartment	post prox
posterior distal	problem	distal end of tibia bone	post dist

## Abbreviations

The following abbreviations are used in this manuscript:

SPI	Serial Peripheral Interface
PLA	Polylactide
USB	Universal Serial Bus
cpi	counts per inch
pcb	printed circuit board
GC	gait cycle
pd	proximodistal
ap	anteroposterior
ml	mediolateral
P2P	Peak-to-Peak
ant prox	anterior proximal measurement site
ant dist	anterior distal measurement site
lat dist	lateral distal measurement site
lat mid	lateral middle measurement site
lat prox	lateral proximal measurement site
post dist	posterior distal measurement site
post prox	posterior proximal measurement site
